# A Treatise on the Structure, Diseases, and Injuries of the Bloodvessels

**Published:** 1847-07-01

**Authors:** 


I. A Treatise on the Structure, Diseases, and Injuries of the Bloodvessels. By *Edward S. Crisp*, M.R.C.S. 8vo. pp. 350. Plates. Churchill, 1847.

II. Observations on Aneurism and its Treatment by Compression. By *O'Bryen Bellingham*, M.D. 12mo. pp. 180. Churchill, 1847.

Mr. Crisp's Treatise is an expansion of the Essay for which the Jacksonian Prize was adjudged in 1844. The institution of this prize has been eminently conducive to the progress of surgical science. Several of the Essays which have gained it have since become standard works upon the subjects to which they respectively relate, and the names of a considerable number of the prize-holders are now European. Mr. Crisp's work, although not admitting of comparison with those of some of his predecessors on the list in originality or novelty of views, is, nevertheless, a very creditable production; and one of which the general practitioners have reason to be proud, as coming from one of their own body, unprovided with any special opportunities in the shape of public appointments, but possessing great zeal and untiring industry in the pursuit of medical science.

After briefly describing the anatomy of the arteries, (assigning to them *four* separate tunics), and presenting a table of the very varied appearances which those of the chest and abdomen offered in 102 cases of death from different causes, Mr. Crisp goes on to consider the subject of *Arteritis*.

He observes very truly, that this has been but little investigated, in consequence of the hasty and imperfect manner in which *post-mortem* examinations are performed. In the case of the *Aorta*, the signs of its inflammation are so various, and its complication with-heart disease so frequent, as generally to defy accurate diagnosis. The only morbid appearances upon which we can depend are more or less permanent reddening, and a pulpy and swollen appearance of the inner and middle tunics—deposits of lymph being also occasionally found in the inner coat. In a few cases the abdominal aorta has been found obliterated by fibrinous coagula. "Although these coagulations are probably in some instances the effects of inflammation, they may, I think, occur from deficiency of the heart's action, combined with a roughened and diseased state of the coats of the aorta. Some of these obstructions may take place a short time before death when the *vis a tergo* is not sufficient to propel the blood through the diseased tube. John Hunter says that the blood will not coagulate in a healthy artery, an opinion which I believe to be correct." P. 29.

When the inflammation attacks the *arteries of the extremities*, its diagnosis is less obscure. Burning, shooting, or pricking pain and tenderness in the course of the vessels are often followed by absence of their pulsation, and with a disposition to dry gangrene of the limb affected. In the aged the inflammation is more subacute, but the tendency to gangrene is greater. A common pathological effect is the obstruction, or even obliteration, of the calibre of the vessel by adherent fibrinous deposits—these extending even sometimes to the collateral and terminating branches.

"Some high authorities believe that arteritis is not present in the dry gangrene of old people. I have paid much attention to this complaint for many years and have had the opportunity of inspecting the bodies of several who have died of it, I have invariably found the lining membrane of the artery of a red appearance, the vessel often obstructed by fibrinous coagulum, some distance above the gangrenous part, as well as in the arteries near the seat of the disease. * * * * * * *

"Many who, like Dr. Carswell, believe that dry gangrene is the result of debility and not of inflammation, appear to have forgotten that inflammatory diseases are very common in old persons. According to the researches of MM. Hourman and Dechambre at the Salpêtrière, inflammation is the most frequent cause of death in old persons. How often the aged are affected with cutaneous inflammation of the legs; and reasoning from analogy, why, let me ask, may not the internal coats of the arteries be subject to the same engorgements? We have, I think, abundant evidence to show that ossification alone is not sufficient to produce the disease, as this deposit exists to a great extent in the majority of individuals who have attained advanced age. The most common of the exciting causes of acute arteritis in the aged is, I believe, the detachment of a portion of the bony plate of the artery. The great toe, a part the most exposed to mechanical injury, is often the first affected." P. 34.

In treating arteritis, we must resort to the same measures we employ for combating inflammation of other organs: but in old subjects, in whom the disease attacks vessels already much altered in structure by bony deposition, Mr. Crisp has found most benefit from the employment of large doses of opium, combined with a mild, non-stimulant diet. When gangrene has supervened, a line of demarcation having formed, the patient being young, and the collateral branches sufficiently enlarged, Mr. Crisp believes amputation is indicated.

Several cases, which have occurred, either in the author's own practice or in that of his friends, some of which are of a highly interesting character, are related in illustration of the above opinions. That dry gangrene, occurring in the young and robust, is very commonly produced by arteritis may be readily allowed; but we doubt very much whether the same term should be applied to the perverted action of the already diseased vessels of the aged. After all, the enquiry is merely a speculative one, for neither the author, or any one else, we suppose, would be willing to treat the gangrene occurring in these subjects antiphlogistically.

*Arterial Deposits.*—These, Mr. Crisp believes, are always situated in the subserous tunic, and not in the fibrous coat, as generally stated. The innermost membrane may often be easily removed, it may be broken by the large bony or atheromatous deposits beneath it, but it is never found ulcerated. The fibrous coat may be reddened or softened by inflammatory action, and in old age it is preternaturally dry, but the deposits are not seated within it. It is liable to hypertrophy or attenuation, without change of structure. Mr. Crisp refers to the frequent occurrence of, in *young subjects,* numerous small spots of opaque white deposits along the origins of the *intercostal arteries,* the membranes around the spots being seldom altered in colour. The *ossification of the arteries* of old persons is generally preceded by an atheromatous or cartilaginous condition of the portions of the tubes affected.

"Writers on this subject appear to think that, if bony deposition takes place to any extent, the elastic property of the vessel must be entirely destroyed. This error has probably arisen from the inspection of dry specimens, in which the patches become consolidated, and sometimes overlap each other. If the arteries be examined soon after death, the intervening substance will be found to retain its elasticity. The seat of these deposits is various, and large portions of the artery may be perfectly free from them, whilst others contain them in great abundance. I have recently examined the body of a woman, 84 years of age, in whom the ascending aorta was in a normal state; but the abdominal and thoracic portions were covered with bony patches. Many persons have lived to a great age, in whom these lesions have existed to a great extent. I think, whether this condition is the effect of inflammation or otherwise, it may be often looked upon as the result of the wear and tear which the arteries, like the other parts of the body, are subjected to; and probably it is a wise provision of Nature to accommodate the circulatory apparatus in old age to the lessened supply of blood required by the secretory organs. These depositions are comparatively infrequent in the young, although instances are recorded of their occurrence at an early period of life." P. 73.

Although the elastic property of the arteries containing osseous deposit may not be entirely destroyed, it must manifestly become much and proportionally diminished. Indeed, Mr. Crisp refers the frequent rupture of the cerebral arteries, and the consequent effusion of blood in the neighbourhood of the corpora striata, to the diminution of their elasticity, by reason of the cartilaginous or atheromatous deposits consequent on chronic inflammation. He suggests, also, that a diseased and consequently obstructed state of the cerebral arteries, may give rise to an impaired condition of the functions of the eye and ear.

After detailing several interesting cases of Disease of the Aortic Valves and of Abdominal Pulsation, the author proceeds to the principal subject, that of—

ANEURISM.

Mr. Crisp is of opinion, as the result of an extensive investigation of morbid specimens, that the *fibrous* coat of the artery is that first affected. He objects to the present terms designating the different kinds of aneurism, but we doubt whether those he suggests will prove very acceptable. "If the term endogenous were substituted for that of spontaneous, when speaking of aneurisms which arise from lesions of the inner coats; and the term exogenous or traumatic applied to those which are produced by external division of the arterial walls, I think the nomenclature would be more appropriate. The various names before alluded to might still be retained to distinguish the varieties of aneurism." After referring to the statements of various authors upon the subject of the proportionate liability of the different arteries to aneurism, he furnishes us with a summary of an extensive table—a most useful *catalogue raisonnée*—which he has constructed of all the cases of aneurism (551) that have been recorded in the British periodicals from 1785 to the present time.

| | | | |
|---|---|---|---|
| Thoracic Aorta | 175 | Innominata | 20 |
| Pulmonary | 2 | Carotid | 25 |
| Abdominal Aorta & Branches | 59 | Cerebral | 7 |
| Common Iliac | 2 | Temporal | 1 |
| External Iliac | 9 | Ophthalmic | 1 |
| Glutæal | 2 | Subclavian | 23 |
| Femoral | 66 | Axillary | 18 |
| Popliteal | 137 | Subscapular | 1 |
| Posterior Tibial | 2 | Brachial | 1 |

Total, 551.

The influence of *sex* is very great, the much larger proportion of males arising, the author believes, from the nature of their occupations. In women, however, whose necks are so much exposed, the occurrence of carotid aneurism is nearly as frequent as in men, viz. 12 to 13 cases. In the whole 551 cases the proportion of females is rather less than one-eighth. In the 243 aneurism of the aorta, pulmonary and cerebral arteries, it amounts to about a fifth. In the 308 cases of external aneurism there are 276 males to 32 females; but in 21 examples of dissecting aneurism the females amount to 14, the males to 7. The disease usually occurs between 30 and 50 years of *age* (it did so in 327 of the cases), and after 60 it becomes comparatively rare. Although the usual *predisposing cause* is supposed to be a diseased condition of the arterial coats, Mr. Crisp believes that it may result from injury done to an artery perfectly free from disease during muscular efforts. The most common of the *exciting causes* of aneurism, indeed, whether internal or external, are violence and sudden muscular effort. The disease seems to be of much more frequent occurrence in England than elsewhere, which the author is disposed to attribute to the more laborious exertions employed by the inhabitants of this country, and their great addiction to the use of spirituous liquors. Sailors and soldiers, and especially the former, are particularly liable to it. Agricultural labourers, owing probably to their more temperate lives, are comparatively free from it. According to the accounts Mr. Crisp has been able to obtain, aneurism seems to be of very rare occurrence in the East and West Indies.

The author considers the various forms of Internal and External Aneurism in full detail, and we regret that we cannot follow him ; but we devote the remainder of the space at our disposal to the important subject of—

*The Treatment of Aneurism by Compression.*

Of this, the greatest improvement of modern times in this portion of surgery, Mr. Crisp gives us but a defective account, furnishing no indication of acquaintance with the principles of its employment, which distinguish it from the mere revival of a practice heretofore frequently attempted and abandoned, For the suggestion, development, and verification of these, we are indeed solely indebted to Dr. Bellingham ; and we feel surprised that Mr. Crisp has omitted all mention of the great credit due to him. It is true, he says, that we are indebted to the "Dublin Surgeons" for the revival of the procedure ; but this statement is too general, many of the Dublin surgeons being just as ignorant of the true principles of its application as those of other places. To the prevalence of the erroneous opinion that the present procedure is a mere revival of former ones, which from their inefficiency fell into disuse, we must attribute the extraordinary remissness which the general body of surgeons have shown in putting it into force. Dr. Bellingham's re-publication, in an enlarged form, of his essay upon the subject, formerly favourably but briefly noticed in this Review, will doubtless do much to remove the misconceptions which prevail upon the subject.

Compression, as originally employed, was directed over the aneurism itself, and was even then successful in comparison to the old operation, which consisted in cutting into and clearing the sac of its coagula, and then tying the vessel above and below the opening—an operation so fatal, that Pott and the best surgeons preferred amputation to it. Compression so employed was however too painful, tedious, and uncertain, to obtain other than occasional success ; and the greater simplicity and safety of the Hunterian operation naturally diverted attention from attempts at improving the mode of its application. The failure of this in some cases was the means of reviving attempts at cure by compression, with the important improvement of directing this over a healthy portion of the artery, instead of the aneurism. Owing, however, to the objects of the pressure being supposed to be the total arrest of the stream of blood through the aneurism, and the securing adhesion of the parietes of the vessel as from the ligature, the compression was so painful and so continuous that few could endure it ; and although a very few cases were thus treated with success in France, and one or two in Dublin, it was not until 1843 the procedure met with any favour here. From this period, however, cases have multiplied, so that Dr. Bellingham is now enabled to present us with the accounts of 27 in which compression has been employed. This gentleman first showed that the degree of compression need not be so excessive as once supposed ; and that it may be made bearable by applying two separate instruments to the limb, and compressing the vessel alternately with one of these, while the other is relaxed. He first furnished the true rationale of the employment of compression. The following are among his observations, made at the Surgical Society of Ireland in 1843.

" It would appear that it is not at all essential the circulation through the vessel leading to the aneurism should be completely checked, but rather the contrary; it may perhaps be advantageous at first, for a short period, by which the collateral circulation will be more certainly established; but the result of this case, if it does no more, establishes the fact, that a PARTIAL CURRENT *through an aneurismal sac will lead to the deposition of fibrine in its interior, and cause it within a few hours to be filled and obstructed, so as no longer to permit of the passage of blood through it.* Pressure, so as altogether to obstruct the circulation of an artery, must necessarily be slower in curing an aneurism, as it must, in some measure, act by causing obliteration of the vessel at the part to which the pressure has been applied; whereas, a *partial current through the sac enables the fibrine to be readily entangled in the parietes of the sac in the first instance, and this goes on increasing until it becomes filled;* the collateral branches having been previously enlarged, the circulation is readily carried on through them." P. 128.

If the process Nature adopts in the *spontaneous cure of aneurism* had been more attentively watched by surgeons, they might sooner have arrived at these conclusions. It will be found she effects this by depositing fibrine in concentric layers, for which a current of blood through the sac is essential. Writers speak of the *deposition of fibrine* and the *coagulation of blood* in the sac as if these were one and the same thing; whereas, both their physical characters and the requisites for their production are quite different. For the production of coagulation a degree of compression which prevented all ingress of blood would be requisite; while fibrine is separated whenever the circulation through the tumour is only diminished. Even the *ligature,* when placed at a distance from the aneurism, operates by producing the diminution of the current through the sac favourable to deposition. If coagulation only took place, which indeed is the case when the ligature is applied close to the aneurism, we should much oftener see suppuration of the sac or secondary aneurism produced.

"Everything which has now been said makes it probable that the ligature of the artery at a distance from the sac, and compression of the vessel at the cardiac side, effect the cure of the aneurism in the same way. It has already been shown that the mode in which Nature brings about a spontaneous cure is precisely similar; which of itself is a strong argument for its correctness. But there is another circumstance which tends still further to confirm it, viz. that several phenomena connected with the surgical treatment of aneurism which were hitherto obscure and inexplicable, upon the theory that the coagulation of the contents of the sac always followed the operation by ligature, are readily explained by this theory. For instance, we have seen that a slight pulsation is not unfrequently felt in the sac subsequent to the operation. Now, this theory not only explains its cause, but it shows why it ceases after a short time, and why, instead of being an unfavourable sign, it should rather be regarded as a favourable one. By this theory alone, we can account for the artery being obliterated after the ligature at the point from which the aneurism springs, while it remains pervious between the ligature and sac. Lastly, it enables us to explain why suppuration of the sac should occur after the operation in one case, and not in others apparently similar; and how a secondary aneurism forms in certain cases a long time after the operation, while in general it must be a rare result of the ligature." P. 147.

The last chapter of Dr. Bellingham's little work is occupied with contrasting the advantages compression possesses over the ligature in respect to its greater simplicity, safety, certainty, and permanency, and its applicability in a variety of cases where the operation by ligature would not be considered justifiable, or when the patient refuses to submit to it. He likewise replies to the few objections which have any apparent plausibility; but these are so easily disposed of that we need not cite their refutation. In our opinion, he has conferred a signal benefit upon the art of surgery by his improvement of the mode of employing pressure, and upon the science by his ingenious and philosophic exposition of its operation. We quote his final conclusions:—

"1. The arteries to which compression is applicable being far more frequently the subject of aneurism than those to which it is inapplicable, compression is calculated to supersede the ligature in the great majority of cases.

"2. The cure of aneurism by compression upon the artery between the aneurismal sac and the heart, according to the rules laid down here, is accomplished by the gradual deposition of the fibrine of the blood in the sac, until both the latter and the artery at the part are completely filled. The process is in fact exactly similar to that by which nature effects a spontaneous cure of aneurism.

"3. Such an amount of pressure as would cause inflammation and adhesion between the opposite sides of the artery at the point compressed is never required.

"4. The pressure should not be so great as to interrupt the circulation in the artery at the point compressed; an essential agent in the cure being that a current of blood should pass through the sac.

"5. Compression by means of two or more instruments, one of which is alternately relaxed, is much more effectual than by any single instrument, and in many instances the pressure can be maintained by the patient himself.

"6. The treatment of aneurism by compression does not involve the slightest risk to the patient, and if persevered in cannot fail of effecting a cure.

"7. A cure of aneurism effected by compression, according to the rules laid down here, must necessarily be permanent; and in every case in which a cure has been accomplished, the patients have remained well subsequently.

"8. The femoral artery remains pervious after the cure at the point at which the pressure had been applied, and no morbid change of any kind is to be detected in either the artery or vein at the site of the compression.

"9. When a cure is effected by compression, the vessel is obliterated only at the seat of the aneurism, and the artery at this part is eventually converted into an impervious ligamentous band.

"10. Compression effects the cure of aneurism by more simple and safer means than the ligature, while it is applicable to a number of cases in which the operation is contra-indicated or inadmissible.

"11. Compression is not necessarily a more tedious or more painful method of treating aneurism than the ligature, while it is much more certain, more likely to be permanent, and is free from all danger.

"12. Compression, according to the rules laid down here, has little analogy with the old method which went by this name; and in fact has no greater resemblance to it than the Hunterian operation had to the operation for aneurism which it superseded." P. 181.

We have no space for any further notice of Mr. Crisp's work; but may observe that his account of the various forms of aneurism, and of the accidents, &c. to which arteries are liable, is very complete; but that the portion of the book relating to Diseases of the Veins is exceedingly meagre. Upon the whole, the work is a valuable contribution to surgery. It is illustrated by some well-executed lithographs.

